# Mean-field models for non-Markovian epidemics on networks

**DOI:** 10.1007/s00285-017-1155-0

**Published:** 2017-07-06

**Authors:** Neil Sherborne, Joel C. Miller, Konstantin B. Blyuss, Istvan Z. Kiss

**Affiliations:** 1 Department of Mathematics, School of Mathematical and Physical Sciences, University of Sussex, Falmer, Brighton, BN1 9QH UK; 2 School of Mathematics, Monash University, Melbourne, VIC Australia; 3 School of Biology, Monash University, Melbourne, VIC Australia; 4 MAXIMA, Monash University, Melbourne, VIC Australia; 5 Institute for Disease Modeling, Bellevue, WA 98005 USA

**Keywords:** Epidemics on networks, Non-Markovian transmission and recovery, Mean-field models, 92D30, 37N25

## Abstract

This paper introduces a novel extension of the edge-based compartmental model to epidemics where the transmission and recovery processes are driven by general independent probability distributions. Edge-based compartmental modelling is just one of many different approaches used to model the spread of an infectious disease on a network; the major result of this paper is the rigorous proof that the edge-based compartmental model and the message passing models are equivalent for general independent transmission and recovery processes. This implies that the new model is exact on the ensemble of configuration model networks of infinite size. For the case of Markovian transmission the message passing model is re-parametrised into a pairwise-like model which is then used to derive many well-known pairwise models for regular networks, or when the infectious period is exponentially distributed or is of a fixed length.

## Introduction

The use of mathematical tools to study and understand the spread of infectious diseases is a mature and fruitful area of research. In their 1927 paper Kermack and McKendrick (1927) established the susceptible-infected-recovered (SIR) framework which forms the basis of many models to this day. However, their model assumes that any individual can interact with any other. In reality, in large populations each individual only interacts with a few others, and these connections determine the possible routes of disease transmission. Moreover, studies have found significant heterogeneity in the number of contacts a single individual may have (Pastor-Satorras and Vespignani 2001). The use of graphs or networks to describe these contact patterns represented a major advance in our ability to model more realistic social behaviour. In network-based models individuals are represented by nodes in the network, with edges (or links) encoding the interactions between nodes.

Since the direct analysis of stochastic epidemics on networks is far from trivial, one often relies on deterministic mean-field models that are aimed at approximating some average quantities taken from the stochastic models. Deriving mean-field models can be done in several different ways depending on what one chooses to focus on. For example, considering all nodes and edges in all possible states leads to pairwise models (Keeling 1999; House and Keeling 2011), while considering separately each individual and all possible ways in which it can become infected by its neighbours leads to the message passing (MP) formalism (Karrer and Newman 2010). Focussing on all possible star-like structures, typically defined by a node and all its neighbours, and also taking into account their disease status, yields the so-called effective-degree models (Lindquist et al. 2011). Edge-based compartmental models (EBCM) are based on considering a randomly chosen test node and working out the probability of it staying susceptible, with this probability being then equivalent to the proportion of susceptible nodes in the entire population (Miller et al. 2012). See Danon et al. (2011), Pastor-Satorras et al. (2015) and Kiss et al. (2017) for reviews. All of these models start from the same stochastic model, thus, it is not surprising that some of these models (House and Keeling 2011; Taylor and Kiss 2014; Miller and Kiss 2014; Kiss et al. 2017) are, in fact, equivalent, as we will demonstrate later.

While network models capture contact more accurately, the assumption that the underlying stochastic transmission and recovery processes are memoryless (Keeling and Eames 2005; Volz 2008; House and Keeling 2011) remains restrictive. Of course, memoryless processes are mathematically more tractable and relatively simple to analyse when compared to models where the inter-event times are chosen from distributions other than the exponential. However, when compared to data, these assumptions are often violated. For example, diseases can exhibit unique and non-Markovian behaviour in terms of the strength and duration of infection. In this respect, the distribution of the infectious period is usually better approximated by some peaked distribution with a well defined mean, see e.g. Bailey (1954), Gough (1977), Wearing et al. (2005) and references therein.

The MP method does not rely on these assumptions and is able to predict the average behaviour of an epidemic outbreak with general distributions for the transmission times and the duration of infection, although we still require these be independent. Throughout the paper we will denote these distributions as $\tau (a)$ and *q*(*a*), where *a* is the time since the node became infected, known as the age of infection. Once a susceptible node has been exposed to a transmission event, it becomes infected immediately, while the recovery from the disease grants a lifetime immunity. Using these distributions assumes a homogeneous response to disease; whilst this restriction is not always necessary (see e.g. Wilkinson and Sharkey 2014), it is a common simplification in order to obtain a concise model. However, the main focus of this paper is to explore the flexibility of the EBCM in being able to capture epidemics where the infection and recovery processes are described by general independent distributions.

The rest of the paper is organised as follows. In the following section we introduce the MP method (Karrer and Newman 2010) and show how the epidemic model is constructed. We then go on to present the extension of the edge-based compartmental model (Miller et al. 2012) to SIR epidemics with general but independent distributions for time to transmission and duration of the infectious period. This section also contains the main result of the paper, namely, a full rigorous proof that MP model and the EBCM are equivalent, and hence, that the EBCM provides an exact representation of the average stochastic behaviour on the ensemble of infinite Configuration Model (CM) networks (Bender and Canfield 1978; Molloy and Reed 1995, 1998). Section 3 contains a re-parametrisation of the MP model in the special case of Markovian transmission. This proves to be a useful tool in showing how several well-known models can be derived from the MP model or the EBCM when additional assumptions about the network or recovery process are made. In Sect. 4, we compare numerical solutions of the mean-field models to averaged results from explicit stochastic network simulations. The paper concludes with a discussion of main results and possible directions for future research.

## Model summary

### The message passing (MP) method

In their 2010 paper, Karrer and Newman (2010) introduced the message passing approach to model SIR dynamics on networks. Here, we briefly present the ideas behind their model and its assumptions. Recalling $\tau (a)$ and *q*(*a*) as the densities for transmission and duration of the infectious period one can introduce a new function *f*(*a*)

1 $$\begin{aligned} f(a) = \tau (a) \int _{a}^{\infty } q(x) \, dx, \end{aligned}$$

such that the probability that a node infected at time 0 attempts to transmit the disease to a given neighbour before time *t* is $\int _{0}^{t} f(a) da$, since a neighbour can only transmit the disease if it has not yet recovered. Note that the integration of (1) over all time is the overall probability of an attempted transmission of the disease across a given network edge, commonly known as the *transmissibility* of the disease. This is a quantity which is important in percolation models to determine the epidemic threshold and expected final epidemic size of a major outbreak (Newman 2002; Kenah and Robins 2007).

In order to model the dynamics of disease spread, consider a test node *u*. This node is placed into a cavity state where it can become infected but is not able to transmit the disease to any of its neighbours. This has no effect on the probability of the node being in any given state (Miller et al. 2012). Now consider a node *v* which is a neighbour of *u*; the *message* is the probability that *v* has *not* attempted to transmit the disease to *u* by calendar time *t*, denoted $H^{u \leftarrow v}(t)$. This probability is comprised of two distinct possibilities; the first possibility is that *v* will make no attempt to transmit the disease before age *t*, this is given by $1 - \int _0^t f(a) \, da$. This means that even if *v* is one of the initially infected nodes, it would not attempt to transmit to *u*. Alternatively, it could be that *v* will transmit to *u* at some age $a <t$, but *v* itself was infected at some time $t_1 > t - a$ and has, therefore, not yet attempted to transmit the disease to its neighbour *u*. This requires *v* to have initially been susceptible (with probability *z*) and to have escaped transmission from each of its neighbours until at least time $(t-a)$. On a tree network with no loops, this is exactly $z \int _0^t f(a)\prod _{w \in \mathcal {N}(v)\backslash u} H^{v \leftarrow w}(t-a) \,da$, where $\mathcal {N}(v)$ denotes the set of neighbours of *v*. Hence, combining these two gives

2 $$\begin{aligned} H^{u \leftarrow v}(t) = 1 - \int _0^t f(a) \left[ 1 - z \prod _{w \in \mathcal {N}(v)\backslash u} H^{v \leftarrow w}(t-a) \right] da. \end{aligned}$$

In principle, one could calculate (2) for all edges (in both directions) to find a full solution for the proportion of the population that is susceptible, infected or removed at any time *t*. For example, the probability that *u* is susceptible is the product of $H^{u \leftarrow w}(t)$ across all neighbours $w \in \mathcal {N}(u)$ multiplied by the probability that it was initially susceptible, *z*. On a single fixed finite tree network, solving (2) for all edges will, in fact, yield the exact solution of the stochastic epidemic (Karrer and Newman 2010). The size of such a system of equations would be twice the number of all edges in the network (since both $H^{u \leftarrow v}(t)$ and $H^{v \leftarrow u}(t)$ would need to be calculated).

Throughout this paper we consider unweighted, bi-directional and static networks constructed according to the configuration model (CM). Every node is assigned a number of neighbours, known as its degree, according to a probability distribution $p_k$, known as the *degree distribution*, that describes the probability that a randomly chosen node has degree *k*. Let us now focus on an ensemble of CM networks and consider an average message, $H_1$, instead of considering distinct messages across every edge (Karrer and Newman 2010). For CM networks, as the size of the network tends to infinity, so does the length of the shortest loops, and, therefore, the network becomes locally tree-like. This means that the messages that a node receives from each of its neighbours are independent, and the average message received by the test node *u* is equal to the product of the average message for each neighbour.

The product in (2) is then this $H_1$ raised to the power of the *excess degree* of the node, its degree excluding the edge which connects it to the test node. The following moment generating functions average this product over the degree distribution

3 $$\begin{aligned}&G_0(x) := \sum _k p_k x^k, \quad G_1(x) := \frac{1}{\langle {k} \rangle } \sum _k p_k k x^{k-1}, \nonumber \\&G_2(x) := G_1'(x) = \frac{1}{\langle {k} \rangle } \sum _k p_k k(k-1) x^{k-2}, \end{aligned}$$

where $\langle {k} \rangle =G_{0}^{'}(1)$ is the mean degree. $G_1(x)$ is the generating function for the *excess degree distribution*, since $kp_k/\langle {k} \rangle$ describes the probability that a node reached by traversing a randomly selected edge has $(k-1)$ other contacts (Newman 2002); the mean excess degree is given by $G_2'(1)$. The moment generating function $G_2(x)$ will be used to trace the route of infection in later models. Using $G_1$ to replace the product in (2), the equation for the average message $H_1(t)$ is

4 $$\begin{aligned} H_1(t) = 1 - \int _0^t f(a) \left[ 1 - z G_1(H_1(t - a)) \right] \, da, \end{aligned}$$

with $$H_1(0) = 1$$.

In practice, the trajectory of $H_1(t)$ is found by identifying and then solving a differential or integro-differential equation. For example, the purely Markovian case (i.e. $\tau (a)$ and *q*(*a*) are both exponential distributions), with transmission and recovery parameters $\beta$ and $\gamma$, leads to

$$\begin{aligned} \frac{dH_1}{dt} = \gamma - (\beta + \gamma )H_1(t) + \beta z G_1(H_1(t)), \end{aligned}$$

where, again, *z* is the fraction of the population which was initially susceptible at time $t=0$ (Karrer and Newman 2010). However, the precise form of this equation is not universal, it depends on the particular choice of $\tau (a)$ and *q*(*a*). The proportions of susceptible, infected and recovered individuals at any time *t* are then given, in terms of the message $H_1(t)$, as

5 $$\begin{aligned} \begin{aligned} \langle {S} \rangle (t)&= z G_0(H_1(t)), \\ \langle {R} \rangle (t)&= \int _0^t q(a)\left[ 1 - \langle {S} \rangle (t - a)\right] \, da, \\ \langle {I} \rangle (t)&= 1 - \langle {S} \rangle (t) - \langle {R} \rangle (t). \end{aligned} \end{aligned}$$

The MP model (5) with the average message $H_1$ is exact when the stochastic epidemic is considered on the ensemble of infinite CM networks (Karrer and Newman 2010). Although this approach is theoretically able to model dynamics for general choices of independent transmission and recovery processes, the need to find a numerically solvable differential equation for $H_1$ in (4) has restricted the use of MP, and numerical examples are limited, see Karrer and Newman (2010), Wilkinson and Sharkey (2014) for several examples where output from the MP model is compared to results based on simulations.

### EBCM for general transmission and recovery processes

The edge-based compartmental model is an established tool for Markovian dynamics (Miller et al. 2012). We introduce a new extended EBCM which generalises the method to general transmission and recovery processes $\tau (a)$ and *q*(*a*). Again, the EBCM uses the fact that the probability that the test node *u* remains susceptible is the probability that *u* escapes transmission from all of its neighbours. This concept is similar to the notion and use of $H_1$ in MP models. Recovery is modelled using age-structured differential equations. However, the EBCM uses the instantaneous rates of transmission and recovery given by the *hazard functions* rather than the raw densities $\tau (a)$ and *q*(*a*). These are defined as

6 $$\begin{aligned} \zeta (a) := \frac{\tau (a)}{\xi _\tau (a)}, \quad \text {and} \quad \rho (a) := \frac{q(a)}{\xi _q(a)}, \end{aligned}$$

where $\xi _\tau (a)$ and $\xi _q(a)$ are the respective *survival functions* (see, e.g., Miller 2011).

7 $$\begin{aligned} \begin{aligned}&\xi _\tau (a) = \int _a^{\infty } \tau (\hat{a}) \, d\hat{a} = e^{\displaystyle {-\int _0^a \zeta (\hat{a})\, d\hat{a}}},\\&\xi _q(a) = \int _a^{\infty } q(\hat{a}) \, d\hat{a} = e^{\displaystyle -\int _0^a \rho (\hat{a}) \, d\hat{a}}. \end{aligned} \end{aligned}$$

All of these disease variables and related functions are summarised in Table 1. As before, the contact network is a CM network with degree distribution and generating functions as defined in (3). The basis of the EBCM revolves around finding the probability that a random test node (in a cavity state) is in a susceptible, infected or recovered state at time *t*. As this test node is chosen at random, these probabilities are equal to the proportions of the population in each state at time *t*, denoted *S*(*t*), *I*(*t*) and *R*(*t*), respectively.

The first important quantity is $\varTheta (t)$, defined in a manner similar to $H_1(t)$ in (4) as the probability that the representative test node has not received transmission from a given neighbour by time *t*. This approach then differs from MP by directly expressing a differential equation for the dynamics of $\varTheta$. The model is known as “edge-based” because it considers the state of the neighbours of the test node; the densities $\Phi _S(t)$, $\Phi _I(t)$ and $\Phi _R(t)$ describe the probability that at time *t* a random neighbour of the test node is (i) still susceptible, (ii) infected but has not attempted to transmit the disease to the test node, (iii) recovered, and did not transmit to the test node whilst it was infected. The age of infection is, in general, crucial in determining the hazard rates, and so we introduce *i*(*t*, *a*) as the density of infected nodes with the age of infection *a*. Similarly, $\phi _I(t,a)$ is the density of infected neighbours who have not transmitted to the test node and have age *a*. Thus, it is clear that $I(t) = \int _0^t i(t,a) da$ and $\Phi _I(t) = \int _0^t \phi _I (t,a) da$. These variables are summarised in Table 2. We also introduce the Dirac delta function as follows Gel’fand and Shilov (1964)

$$\begin{aligned}\delta (x) = {\left\{ \begin{array}{ll} +\infty , \quad x=0 \\ 0, \quad x\ne 0 \end{array}\right. },\int _{-\infty }^{\infty }\delta (x)dx=1. \end{aligned}$$

**Table 1:** The variables and functions describing the transmission and recovery processes

Variable	Definition
$\tau (a)$	The density of the transmission process
*q*(*a*)	The density of the duration of the infectious period
$\xi _{\tau }(a)$	The *survival function* of the transmission process. The probability that an infected node of age *a* has not yet attempted to transmit the disease along a given edge: $\int _a^{\infty } \tau (x) \, dx$
$\xi _{q}(a)$	The *survival function* of the recovery process. The probability that an infected node reaches at least age *a* before recovering: $\int _a^{\infty } q(x) \, dx$
$\zeta (a)$	The *hazard function* of the transmission process. The probability of an edge of age *a* transmitting in a small interval of time $(a, a+\Delta a)$: $\frac{\tau (a)}{\xi _{\tau }(a)}$
$\rho (a)$	The *hazard function* of the recovery process. The probability of an infected node of age *a* recovering in a small interval of time $(a, a+\Delta a)$: $\frac{q(a)}{\xi _{q}(a)}$
*f*(*a*)	The probability that, in a small interval, an infectious contact is made by an infected node of age *a* :$\tau (a)\int _a^{\infty } q(x) \, dx$
*g*(*a*)	The probability that, in a small interval, an infectious node of age *a* recovers, without attempting to transmit the disease to a given neighbour: $q(a)\int _a^{\infty } \tau (x) \, dx$

**Table 2:** The list of variables in the EBCM

Variable	Definition
$\varTheta (t)$	The probability that the initially susceptible test node has not received a transmission from a random neighbour by time *t*
$\Phi _S(t)$	The probability that a random neighbour of the test node *u* is still susceptible
$\Phi _I(t)$	The probability that a random neighbour of the test node *u* is infected, but has not transmitted to *u*
$\phi _I(t,a)$	The probability a random neighbour of the test node *u* to be infected, have not transmitted to *u* by time *t* and have age of infection *a*, $\Phi _I(t) = \int _0^t \phi _I(t,a) \, \mathrm {d}a$
$\Phi _R(t)$	The probability a random neighbour of the test node *u* has been infected and recovered without transmitting to *u*
*S*(*t*)	The density of susceptible nodes
*I*(*t*)	The density of infected nodes
*i*(*t*, *a*)	The density of infected nodes with age of infection *a*
*R*(*t*)	The density of recovered nodes
$G_1(x)$	The generating function of the excess degree distribution: $\frac{1}{\langle {k} \rangle }\sum _{k=0}^\infty p_k k x^{(k-1)}$
$G_2(x)$	The derivative of the generating function of the excess degree distribution: $\frac{1}{\langle {k} \rangle }\sum _{k=0}^\infty p_k k(k-1) x^{(k-2)}$

Before writing down the new edge-based compartmental model it is worth introducing and explaining the structure of its equations. The message $\varTheta$ is monotonically decreasing, and it depends on the density and age of infected neighbours, $\phi _I(t,a)$, and the hazard rate $\zeta (a)$. We must consider the possibility of an infected neighbour of any age up to time *t* transmitting the disease, hence, we have

$$\begin{aligned} \frac{d {\varTheta (t)}}{d {t} } = -\int _0^t \zeta (a) \phi _I(t,a) \, da. \end{aligned}$$

Given $\varTheta (t)$ it follows that $\Phi _S(t) = zG_1(\varTheta (t))$. Since the test node is in a cavity state, the probability of a neighbour being susceptible is the probability of it escaping infection from its other contacts averaged over the excess degree distribution.

The rate at which susceptible neighbours are infected is the boundary condition for infected neighbours, i.e.

$$\begin{aligned} \phi _I(t,0)= - \dot{\Phi }_S(t)= (1-z)\delta (t) + zG_2(\varTheta (t)) \int _0^t \zeta (a) \phi _I(t,a) \, da, \end{aligned}$$

where the first term represents the introduction of the disease at time $t=0$, and is zero everywhere else. As these neighbours age, they may attempt to transmit the disease, and will eventually recover from it. These events depend on the calendar time and the age of infection, and so we have a von Foerster-type equation

$$\begin{aligned} \left( \frac{\partial {}}{\partial {t}} + \frac{\partial {}}{\partial {a}} \right) \phi _I(t,a) = - \left[ \zeta (a) + \rho (a)\right] \phi _I(t,a), \qquad 0 < a \le t. \end{aligned}$$

The density of nodes in each state depends on $\varTheta$ and $\phi _I$. By the same logic seen in the MP model, the density of susceptible nodes is $S(t) = zG_0(\varTheta (t))$. The rate at which susceptible nodes become infected gives the boundary condition of newly infected nodes. Therefore, infected nodes are replenished according to

$$\begin{aligned} i(t,0) = - \dot{S}(t) = (1-z)\delta (t) + \langle {k} \rangle zG_1(\varTheta (t))\int _0^t \zeta (a) \phi _I(t,a) \, da, \end{aligned}$$

where the first term represents the introduction of the disease. As infected nodes age, they recover according to $\rho (a)$; these dynamics depend on *t* and *a*, and we have a second partial differential equation in the model

$$\begin{aligned} \left( \frac{\partial {}}{\partial {t}} + \frac{\partial {}}{\partial {a}} \right) i(t,a) = - \rho (a) i(t,a), \qquad 0 < a \le t. \end{aligned}$$

These equations together form the EBCM for general but independent transmission and recovery processes,

8a $$\begin{aligned} \frac{d {\varTheta (t)}}{d {t} }&= -\int _0^t \zeta (a) \phi _I(t,a) \, da, \end{aligned}$$

8b $$\begin{aligned} \Phi _S(t)&= zG_1(\varTheta (t)),\end{aligned}$$

8c $$\begin{aligned} \phi _I(t,0)&= - \dot{\Phi }_S(t), \nonumber \\&= (1-z)\delta (t) + zG_2(\varTheta (t)) \int _0^t \zeta (a) \phi _I(t,a) \, d a, \end{aligned}$$

8d $$\begin{aligned} \left( \frac{\partial {}}{\partial {t}} + \frac{\partial {}}{\partial {a}} \right) \phi _I(t,a)&= - \left[ \zeta (a) + \rho (a)\right] \phi _I(t,a), \qquad 0 < a \le t, \end{aligned}$$

8e $$\begin{aligned} \Phi _I(t)&= \int _0^t \phi _I(t,a) \, d a, \end{aligned}$$

8f $$\begin{aligned} \Phi _R(t)&= \varTheta - \Phi _S - \Phi _I,\end{aligned}$$

8g $$\begin{aligned} S(t)&= zG_0(\varTheta (t)), \end{aligned}$$

8h $$\begin{aligned} i(t,0)&= - \dot{S}(t), \nonumber \\&= (1-z)\delta (t) + \langle {k} \rangle zG_1(\varTheta (t))\int _0^t \zeta (a) \phi _I(t,a) \, d a, \end{aligned}$$

8i $$\begin{aligned} \left( \frac{\partial {}}{\partial {t}} + \frac{\partial {}}{\partial {a}} \right) i(t,a)&= - \rho (a) i(t,a), \qquad 0 < a \le t, \end{aligned}$$

8j $$\begin{aligned} I(t)&= \int _0^t i(t,a) \, da, \end{aligned}$$

8k $$\begin{aligned} R(t)&= 1-S(t)-I(t). \end{aligned}$$

The new edge-based compartmental model (8) offers an alternative way to derive a system of equations that are able to characterise the dynamics of an epidemic outbreak. Although it seems more complex than the MP model, the EBCM is perhaps more intuitive, as many of the variables it involves relate directly to densities of nodes in different states and to the transitions between different states. The EBCM has also proven to be quite versatile and easily extendable to account for different scenarios. For instance, Miller et al. (2012) extended the original EBCM for static networks to dynamic networks where edges are deleted, created or rewired. It may be possible to use similar techniques to extend (8) to model diseases spreading through dynamic networks. To our knowledge, the MP model has so far not been extended beyond static networks, although it may be possible.

As the MP model (5) and the non-Markovian edge-based compartmental model (8) are based on the same underlying stochastic epidemic, it is natural to question how accurate and how similar they are. Karrer and Newman (2010) showed that the MP model (5) is exact on the ensemble of CM networks. Therefore, proving that the EBCM and MP model are equivalent will imply that the EBCM is exact under the same circumstances.

### Model equivalence

We now present and prove the main result of this paper, that the edge-based compartmental and MP model are equivalent for any suitable choices of $\tau (a)$ and *q*(*a*).

#### Theorem 1

If $\tau (a)$ and *q*(*a*) are independent, integrable probability density functions, then the MP model (5) and the EBCM (8) are equivalent, and will produce identical trajectories for any shared initial conditions.

#### Proof

The proof consists of showing equivalence for two main elements: the messages for the respective models $H_1(t)$ and $\varTheta (t)$, and the densities of nodes in each state.

We first show that $H_1$ and $\varTheta$ satisfy the same evolution equation. To do this, $H_1(t)$ (4) is differentiated using Leibniz’s rule, which yields

9 $$\begin{aligned} \frac{d {H_1}}{d {t} }&= - f(t) [1-z G_1(H_1(0))] - \int _0^t f(a) \left[ -z G_2(H_1(t-a))\frac{d {H_1(t-a)}}{d {t} } \right] \, da \nonumber \\&= -f(t) [1-z G_1(1)] + \int _0^t f(a) \left[ z G_2(H_1(t-a))\frac{d {H_1(t-a)}}{d {t} } \right] \, da \nonumber \\&= -f(t)(1-z) + \int _0^t f(a) \left[ z G_2(H_1(t-a))\frac{d {H_1(t-a)}}{d {t} } \right] \, da. \end{aligned}$$

The dynamics of $\varTheta$ are governed by the following equation

$$\begin{aligned} \frac{d {\varTheta }}{d {t} } = - \int _0^t \zeta (a) \phi _I(t,a) \, da. \end{aligned}$$

From (8d), one can use the integrating factor $\exp \left( \int _0^a [\zeta ( \hat{a}) + \rho (\hat{a}) ] \, d\hat{a}\right)$ to find

10 $$\begin{aligned} \phi _I(t,a) = \phi _I(t-a,0) e^{\displaystyle -\int _0^a [\zeta (\hat{a}) + \rho (\hat{a})] \, d\hat{a}} \end{aligned}$$

where

$$\begin{aligned} \phi _I(t-a,0) = (1-z) \delta (t-a) - z G_2(\varTheta (t-a)) \frac{d {\Theta (t-a)}}{d {t} }. \end{aligned}$$

**Fig. 1:**
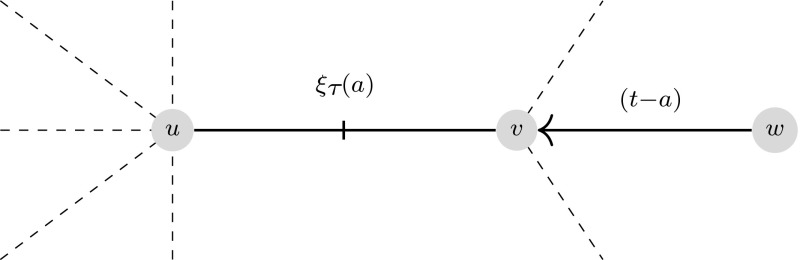
Consider the node labelled *u* as the test node and thus in a cavity state. For its link with node *v* to contribute to $\phi _I(t,a)$, it must be the case that *v* received transmission from some neighbour *w* at time $(t-a)$. If $t-a=0$, then this is equal to the initial proportion of infected nodes. Otherwise, we take the probability of a transmission event *a* time ago, which is $\frac{d {\Theta (t-a)}}{d {t} }$. For *v* to have been successfully infected at this time, it must have been susceptible until that point, since two of its neighbours will not have transmitted before this time (*u* is in a cavity state and *w* will transmit at $(t-a)$). The probability of this is $zG_2(H_1(t-a))$ for $t > a$, illustrated by the dashed lines. The probability of *v* not transmitting to *u* before time *t* is $\xi _{\tau }(a)$. Finally, the neighbour *v* must still be infected at age *a*, which is given by the survival function $\xi _q(a)$

As an alternative, we offer a graphical explanation of (10) in Fig. 1.

Introducing $\hat{f}(a) := \zeta (a) e^{\displaystyle -\int _0^a [\zeta (\hat{a}) + \rho (\hat{a})] \, d\hat{a}}$, we have

11 $$\begin{aligned} \frac{d {\Theta }}{d {t} }&= -\int _0^t \hat{f}(a) \left[ (1-z) \delta (t-a) - z G_2(\Theta (t-a)) \frac{d {\Theta (t-a)}}{d {t} } \right] da \nonumber \\&= - \hat{f}(t) (1-z) + z\int _0^t \hat{f}(a)G_2(\Theta (t-a)) \frac{d {\Theta (t-a)}}{d {t} } \, da. \end{aligned}$$

Thus, $H_1$ and $\Theta$ have the same dynamics if one can show that $f(a)=\hat{f}(a)$. From the definition of *f*(*a*) in (1) and using (7), we obtain

$$\begin{aligned} f(a) = \zeta (a) \xi _{\tau }(a) \xi _q(a) = \zeta (a) e^{\displaystyle -\int _0^a [\zeta (\hat{a}) + \rho (\hat{a})] \, d\hat{a}} = \hat{f}(a). \end{aligned}$$

Since $H_1$ and $\varTheta$ have the same initial condition, this implies that $H_1(t)$ and $\Theta (t)$ will exhibit identical dynamics for general but independent transmission and recovery processes. As a direct consequence, this means that the dynamics of susceptibles are the same in both models, since *S*(*t*) in (8g) and $\langle {S} \rangle (t)$ in (5) differ only in that $\Theta$ is replaced by $H_1$, which are identical and thus interchangeable.

All that remains is to show that the evolution equations for $\langle {I} \rangle (t)$ in (5) and ${\langle I \rangle }{\langle t \rangle }$ in (8j) are identical. From the EBCM we have that

$$\begin{aligned} I(t) = \int _0^t i(t,a) \, da, \end{aligned}$$

which can be differentiated with respect to *t*

$$\begin{aligned} \frac{d {I}}{d {t} }&= \int _{0}^t \frac{\partial {i(t,a)}}{\partial {t}} \, da + i(t,t) \\&= -\int _{0}^t \frac{\partial {i(t,a)}}{\partial {a}} \, da - \int _{0}^t \rho (a)i(t,a) \, da + i(t,t) \\&= i(t,0) - \int _{0}^t \rho (a)i(t,a)\, da, \end{aligned}$$

making use of (8i). The density of infected individuals of age *a* is the same as the density of infected individuals created at time $t-a$ multiplied by the survival probability for the duration of infection, $\xi _q(a)$, i.e.

$$\begin{aligned} i(t,a) = \xi _q(a) i(t-a,0) = \xi _q(a) \dot{S}(t-a). \end{aligned}$$

Utilising this substitution and the second identity in (6) gives

12 $$\begin{aligned} \frac{d {I}}{d {t} } = -\frac{d {S}}{d {t} } - \int _0^t q(a) \frac{d {S(t-a)}}{d {t} } \, da. \end{aligned}$$

From the MP model (5) we can see that

13 $$\begin{aligned} \frac{d {\langle {I} \rangle }}{d {t} }&= -\frac{d {\langle {S} \rangle }}{d {t} } - \frac{d {\langle {R} \rangle }}{d {t} } \nonumber \\&= -\frac{d {\langle {S} \rangle }}{d {t} } -\int _0^t q(a) \frac{d {\langle {S} \rangle (t-a)}}{d {t} } \, da - q(t)(1-z). \end{aligned}$$

This final term describes the rate at which the initially infected individuals recover; this term is implicitly considered in the EBCM in (8h) and so $dI/dt \equiv d\langle {I} \rangle /dt$. This completes the proof. $\square$

An implicit analytical relation for the final epidemic size can be found directly from (8). This result corresponds to well-known results based on tools from percolation theory (Newman 2002; Kenah and Robins 2007; Miller 2007), and to the final epidemic size obtained for MP models (Karrer and Newman 2010). It is given in the following corollary.

#### Corollary 1

The final size of the epidemic $r_\infty = R(\infty )$ in the EBCM (8) with a vanishingly small proportion of infected nodes at time $t=0$ is given by

$$\begin{aligned} r_\infty = 1- G_0(\Theta _\infty ), \end{aligned}$$

where $\Theta _\infty$ solves the equation

$$\begin{aligned} \Theta _{\infty } = 1-\widetilde{T} + \widetilde{T} G_1(\Theta _{\infty }), \end{aligned}$$

and $\widetilde{T} = \int _0^{\infty } f(a) \, da =\int _0^\infty \zeta (a) \exp (-\int _0^a \left[ \zeta (\hat{a}) + \rho (\hat{a}) \right] \, d\hat{a})\, da$ is the transmissibility of the disease, i.e. the probability that the disease is transmitted along an edge (in isolation) before recovery.

#### Proof

From (8g), for $z \rightarrow 1$ and $t \rightarrow \infty$, it immediately follows that

$$\begin{aligned} r_{\infty } = 1 - S(\infty ) = 1 - G_0(\Theta (\infty )). \end{aligned}$$

Furthermore, we have

$$\begin{aligned} \Theta (\infty ) = 1- \int _0^\infty \int _0^t \zeta (a) \phi _I(t,a) \, da \, dt. \end{aligned}$$

Interchanging the order of integration yields

$$\begin{aligned} \Theta (\infty ) = 1 - \int _0^\infty \int _a^\infty \zeta (a) \phi _I(t,a) \, dt \, da. \end{aligned}$$

Setting $u=t-a$ and noting from (10) that $\phi _I(t,a) = \phi _I(u,0)\exp \big (-\int _0^a\big [\zeta (\hat{a})+\rho (\hat{a})\big ]\, d\hat{a}\big )$ yields

$$\begin{aligned} \Theta (\infty )&= 1 - \int _0^\infty \int _0^\infty \phi _I(u,0) \zeta (a) e^{-\int _0^a \left[ \zeta (\hat{a})+\rho (\hat{a})\right] \,d\hat{a}} \, du \, da\\&= 1- \left[ \int _0^\infty \phi _I(u,0) du\right] \int _0^\infty \zeta (a) e^{-\int _0^a \left[ \zeta (\hat{a})+\rho (\hat{a})\right] \, d\hat{a}}\, da\\&= 1+ \left[ \int _0^\infty \dot{\Phi }_S(u) \, du\right] \int _0^\infty \zeta (a) e^{-\int _0^a \left[ \zeta (\hat{a})+\rho (\hat{a})\right] \, d\hat{a}} \, da\\&= 1 + (\Phi _S(\infty )-\Phi _S(0) ) \widetilde{T}\\&= 1 + \widetilde{T} G_1(\Theta (\infty )) -\widetilde{T}. \end{aligned}$$

$$\square $$

Now that the equivalence between the edge-based compartmental and MP models has been established, we consider the special cases resulting from making extra assumptions about the network (e.g. fully connected and regular) and the infection (e.g. Markovian) and recovery processes (e.g. Markovian or infectious periods of fixed length). This is motivated by the observation that many earlier epidemic models are based on $\tau (a)$, *q*(*a*) and/or $p_k$ having the specific forms listed above.

In the following section we aim to produce a model hierarchy by recasting/reducing the EBCM or MP models to earlier models. However, it is not straightforward to see how such earlier models can be derived directly from the EBCM or MP model. This problem can be solved by a re-parametrisation of the MP model in the spirit of pairwise models, and, as a result, one can begin to build a hierarchy of models starting from the most general formulation.

## Model hierarchy

Different model families (pairwise, effective degree, MP, EBCM etc.) emerge from different considerations of the same underlying stochastic process. In this section we aim to produce a model hierarchy on CM networks by showing that for specific choices of network topology or recovery process, many well-known models can be derived from the more general MP model. In particular, we will focus on deriving pairwise models (Wilkinson et al. 2017; Volz 2008; Keeling and Grenfell 1997; Kiss et al. 2015). In order to do this, we first present a general re-parametrisation of the MP model (5), and this will act as stepping stone or interpolation between the EBCM/MP and the well-known earlier models. Since all earlier models use a Markovian infection process, the re-parametrisation also uses this assumption.

Pairwise models are based on differential equations for the expected number of nodes in each state. These depend on the number of edges connecting susceptible and infected nodes, and so differential equations are constructed for the expected number of such edges, which themselves depend on the numbers of triples in certain states (e.g. susceptible-susceptible-infected). To break this dependence, a moment closure approximation is commonly used to express the number of triples in terms of pairs and individuals (Keeling 1999).

Recently, Wilkinson and Sharkey (2014) and Wilkinson et al. (2017) have shown that for regular tree networks exact pairwise models can be derived from the MP model when the transmission process is assumed to be Markovian. Here we use similar methods and the notation from Sect. 2.1 to extend this result to the class of CM networks.

Firstly, we define the new variable $\langle {SI} \rangle (t)$ as the proportion of edges in the network which connect a susceptible node to an infected one at time *t*. This can be defined in terms of existing quantities from the MP model. The susceptible node must have been initially susceptible and escaped infection from all other neighbours until time *t*, given by $zG_1(H_1(t))$. This must be multiplied by the probability that the remaining neighbour of the susceptible node is infected and has not yet transmitted the disease to this neighbour. To find this probability it is easier to calculate all other possibilities and subtract them from one. These possibilities are: (i) the neighbour is still susceptible, (ii) the neighbour has already transmitted the disease, (iii) the neighbour was infected but has recovered without transmitting the disease. Combining these gives

14a $$\begin{aligned} \langle {SI} \rangle (t)&= z G_1(H_1(t))\left[ \phantom {\int _0^t} 1 - zG_1(H_1(t))\right. \nonumber \\&\quad \left. - \int _0^t \{f(a) + g(a) \}\left[ 1 - z G_1(H_1(t - a))\right] \,da\right] , \end{aligned}$$

14b $$\begin{aligned}&= z G_1(H_1(t))\left[ \phantom {\int _0^t} H_1(t) - zG_1(H_1(t)) \right. \nonumber \\&\quad - \left. \int _0^t g(a)\left[ 1 - zG_1(H_1(t-a))\right] \, da\right] , \end{aligned}$$

where

15 $$\begin{aligned} g(a) := q(a)\int _{a}^{\infty } \tau (x) \, dx, \end{aligned}$$

is the probability of an infected node recovering in the interval $(a, a + \Delta a)$ without transmitting to a given neighbour. The corresponding population-level quantity is given by

16 $$\begin{aligned}{}[SI](t) = \langle {k} \rangle N \langle {SI} \rangle (t), \end{aligned}$$

where *N* denotes the total size of the population.

The transmission process is $\tau (a) = \beta e^{-\beta a}$ which can be substituted into (4). Introducing the change of variable $t' = t - a$, and using the Leibniz rule gives

17 $$\begin{aligned} \frac{d {H_1}}{d {t} }&= -\beta \left[ 1 - z G_1(H_1(t)) - \int _0^t q(t - t')e^{-\beta (t-t')}\left[ 1 - zG_1(H_1(t'))\right] \, da \right. \nonumber \\&\quad - \left. \int _0^{t} \beta e^{-\beta (t - t')} \left( \int _{t - t'}^{\infty } q(x) dx\right) \left[ 1 - zG_1(H_1(t'))\right] \, dt' \right] \nonumber \\&= -\beta \left[ 1 - zG_1(H_1(t)) - \int _0^t \{f(a) + g(a) \}\left[ 1 - zG_1(H_1(t-a))\right] \, da\right] \nonumber \\&= -\beta \frac{\langle SI \rangle (t)}{z G_1(H_1(t))} \end{aligned}$$

18 $$\begin{aligned}&= -\beta \frac{[SI]}{z \langle {k} \rangle N G_1(H_1(t))}. \end{aligned}$$

For the infected population, using (5) and identities, such as $[S](t) = N\langle {S} \rangle (t)$, leads to

19 $$\begin{aligned} \dot{[I]}&= - \dot{[S]} - \dot{[R]} \end{aligned}$$

20 $$\begin{aligned}&= \beta [SI] - \beta \int _0^t q(a) [SI](t-a) da - q(t)N(1-z). \end{aligned}$$

The majority of pairwise epidemic models retain an explicit differential equation for the infected population (House and Keeling 2011; Wilkinson et al. 2017). However, we choose to integrate (20) to reduce the number of differential equations that have to be integrated numerically. By noting that $[SI] = \beta \int _0^t \dot{[SI]}(t-a)\xi _q(a) \, da$ and $q(a) = -\xi _q'(a)$, we have

$$\begin{aligned} \dot{[I]} = \beta \int _0^t \left( \dot{[SI]}(t-a)\xi _q(a) + \xi _q'(a) [SI](t-a) \right) \, da - q(t)N(1-z), \end{aligned}$$

which is the result of differentiating

21 $$\begin{aligned}{}[I] = \beta \int _0^t [SI](t-a)\xi _q(a) \, da + N(1-z)\xi _q(t). \end{aligned}$$

Whilst (20) facilitates easier comparison to existing models, its equivalent representation (21) offers greater computational efficiency.

For the variable $\langle SI \rangle$ the calculation is more laborious; working term-by-term from (14b) and using the new relation (18) one obtains

$$\begin{aligned} \dot{\langle SI \rangle }&= -z G_2(H_1)\left( \beta \frac{[SI]}{z \langle {k} \rangle N G_1(H_1(t))}\right) \Bigg [\cdot \Bigg ] \\&\quad - \beta \frac{[SI]}{\langle {k} \rangle N} + z \beta [SI] \frac{G_2(H_1(t))}{\langle {k} \rangle N} - q(t)e^{-\beta t}(1-z)z G_1(H_1(t)) \\&\quad - z \beta \int _0^t q(a)e^{-\beta a}[SI](t-a)G_2(H_1(t-a)) \frac{G_1(H_1(t))}{N\langle {k} \rangle G_1(H_1(t-a))} \, da, \end{aligned}$$

where $[\cdot ]$ denotes the large bracket in (14b), and the Leibniz rule has been used again to resolve the integral term. Finally, based on (14), $[\cdot ]=\frac{\langle SI \rangle }{zG_{1}(H_1)}$, which allows us to eliminate $[\cdot ]$ and replace it with a term involving $\langle SI \rangle$. Then, multiplying through by $\langle {k} \rangle N$ one obtains $\dot{[SI]}$ in (22a) below.

22a $$\begin{aligned} \dot{H_1}&= -\beta \frac{[SI]}{z \langle {k} \rangle N G_1(H_1)}, \end{aligned}$$

22b $$\begin{aligned} \dot{[SI]}&= -\beta [SI]^2\frac{G_2(H_1)}{z\langle {k} \rangle N [G_1(H_1)]^2} - \beta [SI] \nonumber \\&\phantom {-\beta } + z \beta [SI]G_2(H_1) - q(t)e^{-\beta t}(1-z)z G_1(H_1) \langle {k} \rangle N \end{aligned}$$

22c $$\begin{aligned}&\phantom {-\beta } - z \beta \int _0^t q(a)e^{-\beta a} [SI](t-a)G_2(H_1(t-a)) \frac{G_1(H_1(t))}{G_1(H_1(t-a))} \, da , \nonumber \\ [I]&= \beta \int _0^t [SI](t-a)\xi _q(a) \, da + N(1-z)\xi _q(t). \end{aligned}$$

At any time *t* the expected number of susceptibles can be found as $[S](t) = zN G_0(H_1(t))$. System (22a) has been derived directly from the MP model, and thus it becomes exact under the same conditions - on the ensemble of CM networks as the network size tends to infinity. Moreover, retaining the concept of the message, $H_1$, has meant that system (22a) does not depend on higher order arrangements of nodes (e.g. triples). Therefore, unlike most pairwise models, no further approximations are required to close the model. Similar results have been achieved in the past using heuristic arguments (House and Keeling 2011).

This re-parametrised system (22a) is the first crucial step in being able to move from general to specific models on CM networks, with special focus on unifying various approaches by considering different models from the same perspective.

**Fig. 2:**
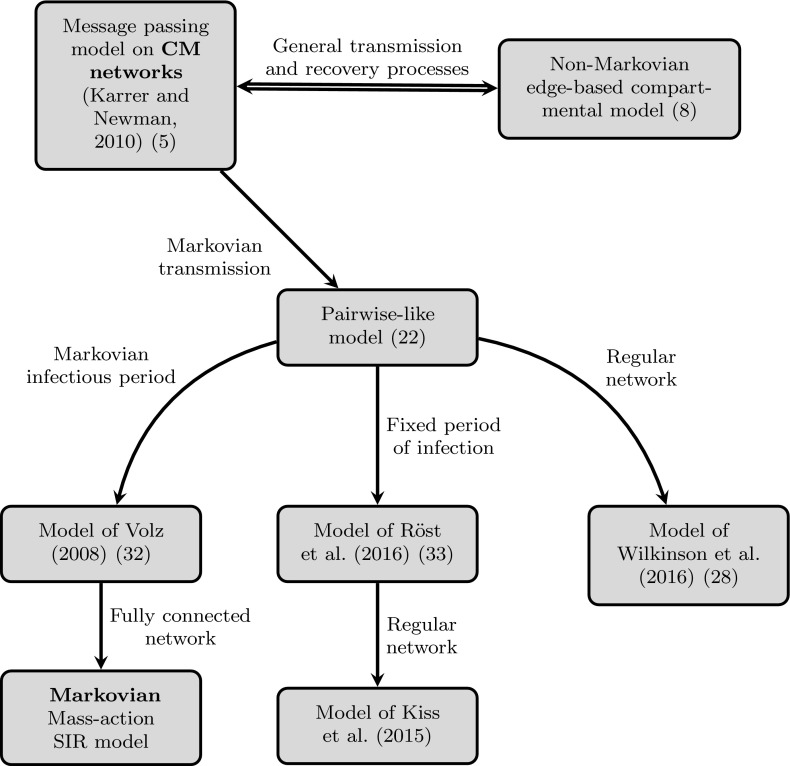
Diagram showing the relationship between the various models discussed in the paper. Labels on each branch state the necessary assumptions in order to reach the model at the end of the *arrow*

As one would expect, earlier population-level models were constructed based on some more restrictive assumptions on network and epidemic dynamics. We will show that when these are applied to system/model (22a), earlier models can be easily recovered. The simplifying assumptions refer either to the network [e.g. fully connected or regular (Wilkinson et al. 2017)], or the distribution of the infectious period [e.g. Markovian (Volz 2008), fixed length (Kiss et al. 2015)]. The remainder of this section is devoted to the explicit derivation of the relationships between models as illustrated in Fig. 2.

### Degree-regular networks

The first of these reductions concerns the special case of regular networks. For a *k*-regular (homogeneous) network, all nodes have the same degree, i.e. $k_u = \langle {k} \rangle = k$, and so the generating functions from (3) simplify to

$$\begin{aligned} G_0(x) = x^k, \quad G_1(x) = x^{k-1}, \quad \text {and} \quad G_2(x) = (k-1) x^{k-2}. \end{aligned}$$

We also introduce two new variables

23 $$\begin{aligned} {\begin{matrix} [S](t) &{}= zN G_0(H_1(t)) = zN[H_1(t)]^k, \\ [SS](t) &{}= \langle {k} \rangle N \left( zG_1(H_1(t))\right) ^2 = kN\left( z[H_1(t)]^{k-1}\right) ^2, \end{matrix}} \end{aligned}$$

that represent the expected number of susceptible individuals and the expected number of edges connecting two susceptible nodes, respectively. [*S*](*t*) follows directly from (5), and [*SS*](*t*) is defined as the number of edges connecting two nodes who were both initially susceptible at time $t=0$ and have escaped infection from their $(k-1)$ other neighbours.

Now we return to the system (22a) and the differential equation for [*I*] (20). Substituting the simpler generating functions yields

24 $$\begin{aligned} \begin{aligned} \dot{H_1}&= -\beta \frac{[SI]}{z k N H_1^{k-1}}, \\ \dot{[I]}&= \beta [SI] - \beta \int _0^t q(a) [SI](t-a) da - q(t)N(1-z), \\ \dot{[SI]}&= -\beta [SI]^2\frac{(k-1)H_1^{k-2}}{zkN [H_1^{k-1}]^2} - \beta [SI] \\&\quad + z \beta [SI](k-1)H_1^{k-2} - q(t)e^{-\beta t}(1-z)z H_1^{k-1} k N\\&\quad - z \beta \int _0^t q(a)e^{-\beta a} [SI](t-a)(k-1)[H_1(t-a))]^{k-2} \frac{[H_1(t)]^{k-1}}{[H_1(t-a)]^{k-1}} da. \end{aligned} \end{aligned}$$

This can be simplified further using (23), firstly noting that

25 $$\begin{aligned} \frac{[SS]}{[S]} = \frac{N k z^2 H_1^{2(k-1)}}{N z H_1^k} = k z H_1^{k-2}, \end{aligned}$$

and, from $\dot{H_1}$ we see that

26 $$\begin{aligned} \frac{d {(H_1^{k-1})}}{d {t} } = -\beta (k-1)\frac{[SI]}{z k N H_1^{k-1}} H_1^{k-2} = -\beta \frac{(k-1)}{k}\frac{[SI]}{[S]} H_1^{k-1}. \end{aligned}$$

Solving for $H_1^{k-1}$ using separation of variables gives

27 $$\begin{aligned} H_1^{k-1}(t) = \exp \left( -\beta \int _0^t \frac{(k-1)}{k}\frac{[SI](a)}{[S](a)}\, da \right) . \end{aligned}$$

The result of this is that the system no longer requires the message $H_1$, as one can calculate the time derivatives of [*S*] and [*SS*] directly from (23). Using the new relations (25) and (27), system (24) can be rewritten to give

28 $$\begin{aligned} \begin{aligned} \dot{[S]}&= - \beta [SI], \\ \dot{[I]}&= \beta [SI] - \beta \int _0^t q(a) [SI](t-a) da - q(t)N(1-z), \\ \dot{[SS]}&= -2\beta \frac{(k-1)}{k}\frac{[SS][SI]}{[S]}, \\ \dot{[SI]}&= - \beta \frac{(k-1)}{k}\frac{[SI][SI]}{[S]} - \beta [SI] + \beta \frac{(k-1)}{k}\frac{[SS][SI]}{[S]} \\&\phantom {-\beta } - k Nq(t)e^{-\beta t}(1-z)z \exp \left( -\beta \int _0^t \frac{(k-1)}{k}\frac{[SI](a)}{[S](a)}da \right) \\&\phantom {-\beta } - \beta \int _0^t q(a)e^{-\beta a} \frac{(k-1)}{k}\frac{[SS](t - a)[SI](t-a)}{[S](t- a)} \mathcal {F}(t)da, \end{aligned} \end{aligned}$$

where

29 $$\begin{aligned} \mathcal {F}(t) = \exp \left( -\beta \int _{t - a}^t \frac{(k-1)}{k}\frac{[SI](u)}{[S](u)} du\right) . \end{aligned}$$

This is identical to the system proposed by Wilkinson et al. (2017). Recently, Röst et al. (2016) have considered the same problem, namely, an SIR epidemic with Poisson transmission and an arbitrary distribution of the infectious period on a regular network. By constructing an age-structured system of PDEs they were able to reach a very similar, albeit a more compact model. We have, therefore, shown that (22a) extends these recent models by allowing general degree distributions to be modelled.

### Special distributions of the infectious period

As mentioned previously, a popular choice for the duration of infection is to assume an exponential distribution, i.e. $q(a) = \gamma e^{-\gamma a}$ for $\gamma >0$, where $1/\gamma$ is the mean duration of infection. We briefly explain how this assumption simplifies the model and leads to familiar or well-known models. When this choice for *q*(*a*) is substituted into (20), we have

30 $$\begin{aligned} \dot{[I]} = \beta [SI] - \gamma \left[ \int _0^t e^{-\gamma a} \beta [SI](t-a) da + e^{-\gamma t}N(1-z)\right] . \end{aligned}$$

Note that $e^{-\gamma a}$ is the probability of an infected node not recovering before age *a*, and since the number of infected nodes created *a* time ago is $\beta [SI](t-a)$ for $a < t$ and $N(1-z)$ for $a=t$, (30) can be rewritten as

31 $$\begin{aligned} \dot{[I]} = \beta [SI] - \gamma [I]. \end{aligned}$$

A similar result is reached in (22b). In this case the extra terms in the integral describe the probability for the susceptible node of an [*SI*] edge to have survived until age *a* without receiving transmission, either along this edge or from another infected neighbour. Therefore, by the same logic one can replace the final two terms in (22b) with $\gamma [SI]$. This leads to a model, which, although formulated differently, is similar to models of Volz (2008) and House and Keeling (2011), namely,

32 $$\begin{aligned} \begin{aligned} \dot{H_1}&= -\beta \frac{[SI]}{z \langle {k} \rangle N G_1(H_1)}, \\ \dot{[I]}&= \beta [SI] - \gamma [I], \\ \dot{[SI]}&= -\beta [SI]^2\frac{G_2(H_1)}{z\langle {k} \rangle N [G_1(H_1)]^2} - (\beta + \gamma )[SI] + z \beta [SI]G_2(H_1). \end{aligned} \end{aligned}$$

If one further assumes that the degree is regular, repeating the steps used to derive system (28) recovers the early pairwise model (Keeling and Grenfell 1997).

We examine one final special case, when the duration of infection is a fixed period of time, $\sigma$, so that $q(a) = \delta (a - \sigma )$. This means that as soon as a node is infected at time $t_1$, it is known that this node will recover at exactly $t_2 = t_1 + \sigma$. Therefore, the integral terms are non-zero only at the point $a = \sigma$. In this case the system of integro-differential equations (22a) simplifies to a delay differential equation model, as stated below

33 $$\begin{aligned} \begin{aligned} \dot{H_1}&= -\beta \frac{[SI]}{z \langle {k} \rangle N G_1(H_1)}, \\ \dot{[I]}&= \beta [SI] - \beta [SI](t-\sigma ) - \delta (t-\sigma )N(1-z), \\ \dot{[SI]}&= -\beta [SI]^2\frac{G_2(H_1)}{z\langle {k} \rangle N [G_1(H_1)]^2} - \beta [SI] \\&\phantom {-\beta } + z \beta [SI]G_2(H_1) - \delta (t - \sigma )e^{-\beta t}(1-z)z G_1(H_1) \langle {k} \rangle N\\&\phantom {-\beta } - z \beta e^{-\beta \sigma } [SI](t-\sigma )G_2(H_1(t-\sigma )) \frac{G_1(H_1(t))}{G_1(H_1(t-\sigma ))}. \end{aligned} \end{aligned}$$

This model generalises the recent work of Kiss et al. (2015) to heterogeneous networks, and once again the original model in that paper can be retrieved when *q*(*a*) is chosen to be a delta distribution in (28) (although that original model did not explicitly account for the recovery of initially infected nodes).

Finally, it is worth briefly noting that in the case of a fully connected network, corresponding to a homogeneously well-mixed population we have that $[SI] = [S][I]$, and thus, the Markovian mass-action SIR model, which assumes that the population is unstructured, is recovered. Moreover, with the proper conditions, Wilkinson et al. (2017) proved that the message passing model is equivalent to the mass action model of Kermack and McKendrick (1927) for general transmission and recovery processes.

## Numerical simulation results

In order to illustrate the accuracy of (22a), we compare the numerical solution of this model to results of direct stochastic network simulation. A common approach for simulating traditional Markovian models has been to use the Gillespie algorithm (Gillespie 1977). However, as modelling started to move away from the purely Markovian models, novel stochastic simulation methods have been derived (Anderson 2007; Boguná et al. 2014) which provide efficient simulation algorithms that are able to generate true sample paths of the stochastic process.

In this section we take advantage of the fact that in the system (22a) transmission is a Poisson process in order to use an algorithm similar to those described by Barrio et al. (2006). This approach is sometimes known as the rejection method and was proven to be stochastically exact by Anderson (2007). The transmission process is run as in the standard Gillespie algorithm; whenever a node becomes infected, a duration of infection is drawn from the distribution *q*(*a*); at each time step the time of next transmission is randomly generated, but if an infected node is scheduled to recover sooner, then the transmission event is rejected, and time is updated to the next recovery time (for full details see Anderson 2007).

In the very early stages of an outbreak stochastic effects dominate the dynamics of the epidemic spread, which means that numerical simulations can often produce results that significantly differ from deterministic predictions. In this situation, methods such as branching process approximations (Heesterbeek 2000) are more appropriate. To ensure that this does not affect our results, we allow every iteration of the algorithm to reach a point where the stochastic effects are no longer a concern, and the infected population behaves deterministically. In practice this is achieved by running each individual realisation of the epidemic from a single initial seed until a specified level of infectivity is reached, at which point time is reset to zero in both the simulation and the mean-field model.

A sufficient number of individual simulations are averaged to ensure that the mean behaviour of the stochastic model is correctly captured and is suitable for comparison with results derived from the deterministic models.

**Fig. 3:**
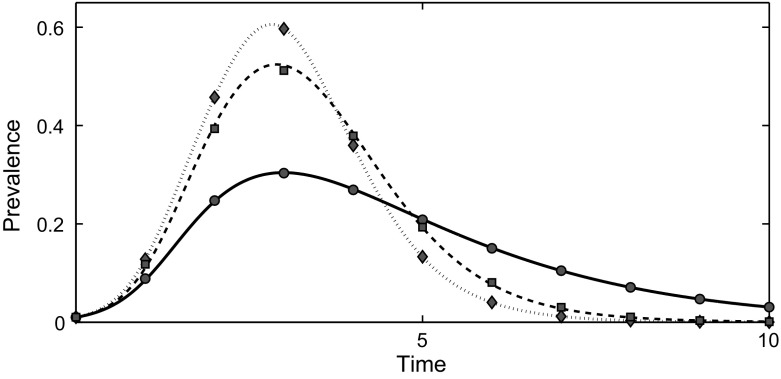
Comparison between system (22a) and the average of numerical simulations. All tests are carried out on randomly generated, truncated scale-free networks of 1000 nodes with exponent 2.5, and the degree bounded between 3 and 60. The transmission rate is set to $\beta =0.3$ in all cases. Results are plotted where the infectious period is exponentially distributed with parameter $\gamma = 0.5$ (*solid line*, *circles*), normally distributed with mean 2 and standard deviation 0.75 (*dashed line*, *squares*), and a fixed duration $\sigma = 2$ (*doted line*, *diamonds*). The mean duration of the infectious period is equal to 2 for all cases. The differences between the epidemics show that the shape of the distribution of the infectious period can have a significant effect on the dynamics of the epidemic

In Fig. 3 the results of numerical simulations are shown for three different distributions of the infectious period all having the same mean: a normal distribution, an exponential distribution, and a fixed infectious period. Two important observations can be made. Firstly, the excellent agreement between the average of simulations (markers) and the mean-field model (lines) provides empirical validation of the mean-field model. Secondly, Fig. 3 shows marked differences between the epidemics despite the mean of the infectious periods being the same. In particular, the exponential distribution leads to the slowest epidemic growth (and smallest epidemic peak) with the infectious periods of fixed length leading to the fastest growing epidemics (and largest epidemic peak). These results highlight the potential risks of using inaccurate modelling assumptions. The results also suggest that the variance in the duration of the infectious period has a significant effect on the time evolution of the epidemic: a decrease in variance leads to an increase in the initial growth rate (Kiss et al. 2015).

Changes to the degree distribution, transmission and infectious processes will all have an impact on the final epidemic size, as determined by Corollary 1. In the tests shown in Fig. 3 only the distribution of the infectious period changes. Whilst this will affect the final epidemic size, it is not possible to tell which of the epidemics is going to produce the largest final epidemic size purely from examining the time evolution of the epidemic in Fig. 3. Indeed, it is possible for two epidemics with different time evolutions to have the same final epidemic size. For example, if the degree distribution remains the same, different choices for the distributions of the time to infection and infectious periods can produce identical values for the transmissibility, and the same final epidemic size.

## Discussion

In this paper we have reviewed the message passing formalism for SIR epidemics on networks, and introduced a novel extension of the edge-based compartmental model to the case of general but independent transmission and recovery processes. Both of these models are capable of accurately describing the expected dynamics of non-Markovian epidemics on tree networks. The main result of the paper is the complete and rigorous of equivalence between these models, and, as a result, the non-Markovian EBCM (8) is exact on the ensemble of infinite-size CM networks.

Adapting recent methods (Wilkinson and Sharkey 2014; Wilkinson et al. 2017) enabled us to re-parametrise the MP model for the special case of a Markovian transmission process but arbitrary CM networks. The compact model (22a) remains exact and is, in fact, a hybrid between MP and classical pairwise models.

Many pairwise models are defined heuristically (Eames and Keeling 2002; Gross et al. 2006; House and Keeling 2011), but by deriving model (22a) as a re-parametrisation of the MP model we have developed a general model from which existing pairwise models can be extracted. By demonstrating this we hope to provide some intuition for how these newer models work and to illustrate that they build on existing models whilst providing a modern twist. It is encouraging that such mean-field models remain relatively compact, highlighting that the SIR epidemic can be modelled quite effectively, as long as a small number of key characteristics of the network and the epidemic process are known.

The results from our numerical simulations illustrate the dangers of using inaccurate or overly simplistic data to make predictions, in particular, the common assumption of fully Markovian dynamics. The MP and non-Markovian edge-based compartmental models are, therefore, crucial if we are to develop accurate epidemic models on networks.

Numerous extensions of the present work are possible. For example, the implementation of an efficient solver of the novel EBCM is still outstanding. Efficient numerical methods to solve such age-structured models exist, but this was outside the scope of our study. In some sense the novel EBCM is the most complete mean-field model when one considers SIR epidemics on CM networks. This is due to the model being able to handle arbitrary degree distributions, as well as general independent transmission and recovery processes. Additionally, it could be refined to account for dynamic or adaptive contacts. Dynamic networks have already been incorporated in edge-based modelling in the purely Markovian setting (Miller et al. 2012), and it may be possible to extend this to a more general framework to allow for a more unified treatment of models that include the concurrent spread of the disease and link turnover.
